# Determining the optimal time for liberation from renal replacement therapy in critically ill patients: a systematic review and meta-analysis (DOnE RRT)

**DOI:** 10.1186/s13054-020-2751-8

**Published:** 2020-02-13

**Authors:** Riley Jeremy Katulka, Abdalrhman Al Saadon, Meghan Sebastianski, Robin Featherstone, Ben Vandermeer, Samuel A. Silver, R. T. Noel Gibney, Sean M. Bagshaw, Oleksa G. Rewa

**Affiliations:** 1 Department of Critical Care Medicine, Faculty of Medicine and Dentistry, University of Alberta, 2-124E Clinical Sciences Building 8440 112 St. NW, Edmonton, Alberta T6G 2B7 Canada; 2 Alberta Strategy for Patient-Oriented Research (SPOR) SUPPORT Unit Knowledge Translation Platform, University of Alberta, 4-472 Edmonton Clinic Health Academy, 11405 – 87 Avenue, Edmonton, Alberta T6G 1C9 Canada; 3 Alberta Research Center for Health Evidence (ARCHE), University of Alberta, 4-496 Edmonton Clinic Health Academy, 11405 – 87 Avenue, Edmonton, Alberta T6G 1C9 Canada; 4 Division of Nephrology, Department of Medicine, Queen’s University, 94 Stuart Street, Kingston, Ontario K7L 3N6 Canada

**Keywords:** Systematic review, Renal replacement therapy, Prediction, Acute kidney injury, Intensive care unit, Biomarkers, Creatinine

## Abstract

**Introduction:**

Renal replacement therapy (RRT) is associated with high mortality and costs; however, no clinical guidelines currently provide specific recommendations for clinicians on when and how to stop RRT in recovering patients. Our objective was to systematically review the current evidence for clinical and biochemical parameters that can be used to predict successful discontinuation of RRT.

**Methods:**

A systematic review and meta-analysis were performed with a peer-reviewed search strategy combining the themes of renal replacement therapy (IHD, CRRT, SLED), predictors of successful discontinuation or weaning (defined as an extended period of time free from further RRT), and patient outcomes. Major databases were searched and citations were screened using predefined criteria. Studied parameters were reported and, where possible, data was analyzed in the pooled analysis.

**Results:**

Our search yielded 23 studies describing 16 variables for predicting the successful discontinuation of RRT. All studies were observational in nature. None were externally validated. Fourteen studies described conventional biochemical criteria used as surrogates of glomerular filtration rate (serum urea, serum creatinine, creatinine clearance, urine urea excretion, urine creatinine excretion). Thirteen studies described physiologic parameters such as urine output before and after cessation of RRT, and 13 studies reported on newer kidney biomarkers, such as serum cystatin C and serum neutrophil gelatinase-associated lipocalin (NGAL). Six studies reported sensitivity and specificity characteristics of multivariate models. Urine output prior to discontinuation of RRT was the most-studied variable, with nine studies reporting. Pooled analysis found a sensitivity of 66.2% (95% CI, 53.6–76.9%) and specificity of 73.6% (95% CI, 67.5–79.0%) for urine output to predict successful RRT discontinuation. Due to heterogeneity in the thresholds of urine output used across the studies, an optimal threshold value could not be determined.

**Conclusions:**

Numerous variables have been described to predict successful discontinuation of RRT; however, available studies are limited by study design, variable heterogeneity, and lack of prospective validation. Urine output prior to discontinuation of RRT was the most commonly described and robust predictor. Further research should focus on the determination and validation of urine output thresholds, and the evaluation of additional clinical and biochemical parameters in multivariate models to enhance predictive accuracy.

## Introduction

Acute kidney injury (AKI) is a common problem encountered in the intensive care unit (ICU), estimated to occur in up to 60% of all critically ill patients, depending on the definition [1]. When AKI progresses in severity, treatment with renal replacement therapy (RRT) may be initiated. RRT is currently applied in 23.5% of ICU patients with AKI (i.e., 13.5% of all ICU admitted to the ICU), with utilization growing by over 10% per year over the past decade [2, 3]. Recent large randomized clinical trials (RCTs) in critical care nephrology have focused on the optimal timing of initiation of acute RRT [4–6]. However, less is known about the ideal circumstances in which RRT may be successfully discontinued. RRT is a complex and expensive therapy, with complications including catheter-associated infections, hemorrhage, hemodynamic instability, and delayed renal recovery [7–15]. It is thus imperative to recognize when a patient may be safely liberated from this treatment. The Kidney Disease Improving Global Outcomes (KDIGO) organization has stated in their 2012 Clinical Practice Guidelines for Acute Kidney Injury that RRT should be discontinued “when it is no longer required, either because intrinsic kidney function has recovered to the point that it is adequate to meet patient needs, or because RRT is no longer consistent with the goals of care.” [16]. However, this recommendation was based on expert opinion and lacks specific guidance for how clinicians should assess patients for suitability to discontinue RRT.

Numerous parameters have been evaluated to help identify patients for whom RRT may be safely discontinued, including traditional biochemical markers of kidney function (creatinine, urea, and estimates of glomerular filtration rate (GFR) [17–26]), clinical findings such as urine output [21–23, 26, 27], and newer kidney biomarkers including neutrophil gelatinase-associated lipocalin (NGAL) [19, 28, 29] and serum cystatin C [28–31]. Despite many criteria being evaluated in the existing literature, the available evidence has yet to be rigorously synthesized. Our objective was to conduct a systematic review and meta-analysis to identify predictors of successful discontinuation of acute RRT among critically ill patients with AKI.

## Methods

We performed a systematic review using methodological approaches outlined in the Cochrane Handbook for Systematic Reviews of Interventions [32] and described according to the Preferred Reporting Items for Systematic Meta-Analyses (PRISMA) guideline [33]. A PRISMA checklist is available as Additional file 1. Research ethics approval was not required. This systematic review was registered at PROSPERO (2018-03-06, CRD42018074615), and the protocol was published separately [34].

### Search strategy

The search strategy was developed in consultation with a research librarian and independently peer-reviewed by a second librarian. We searched electronic databases: Ovid MEDLINE (1946-), Ovid Embase (1988-), and Wiley Cochrane Library (inception-) on October 10, 2017, with an updated search on April 8, 2019. Our search strategy combined concepts related to renal replacement therapy (i.e., intermittent hemodialysis (IHD), slow low-efficiency dialysis (SLED), continuous renal replacement therapy (CRRT)), intensive care (i.e., involving any intensive care unit (ICU) setting), and discontinuation of therapy (i.e., either clinical, physiological, and biochemical parameters of weaning acute RRT) or treatment outcome (Additional file 2). Search results were limited to publications after 1990, when continuous venovenous RRT was initiated. No language limits were applied.

Additional search sources included the trial registry platforms (i.e., ClinicalTrials.gov) and Google Scholar. We also searched meeting abstracts where available using Conference Proceedings Citation Index (Clarivate Analytics) and by hand-searching published proceedings from the following associations and meetings: American Society of Nephrology, Canadian Society of Nephrology, “CRRTonline” (San Diego), European Renal Association – European Dialysis and Transplant Association, European Society of Intensive Care Medicine, International Symposium on Intensive Care and Emergency Medicine (Brussels), National Kidney ?>Foundation, and Society of Critical Care Medicine. Search results were exported and screened in EndNote X7 (Thomson Reuters, Philadelphia, Pennsylvania). See Additional file 2 for the complete search strategy.

### Study selection

Eligible articles were identified through a two-phase process. In the first phase, two authors (AA, RJK) independently reviewed the titles and abstracts of all retrieved articles and documents. Disagreements were resolved through discussion or adjudication by a third author (OGR). In the second phase, full texts of the selected articles were reviewed by the same two authors independently and reviewed for eligibility using standard, predefined criteria. Disagreements were resolved through a discussion with a third author (OGR).

### Eligibility criteria

Studies were included if they mentioned all of the following themes: (1) intensive care (i.e., intended to refer to patients supported in an ICU setting capable of providing invasive mechanical ventilation or vasoactive therapy), (2) renal replacement therapy (i.e., IHD, SLED, CRRT), and (3) described parameters associated with weaning or discontinuation (i.e., clinical, physiological, and biochemical parameters). Additionally, we only included adult patients (i.e., age greater or equal to 18 years old) for this review. Studies which did not mention all of these themes were excluded.

### Risk of bias assessment

Study methodological quality was independently rated by two authors (AA, RK) using the Newcastle-Ottawa Scale (NOS) for observational studies [35]. Observational studies were rated as high quality if they had a total score of 6–9, moderate quality with a score of 4 or 5, and poor quality if the score was 3 or lower. In order to account for potential bias due to population selection in observational trials, the NOS score for comparability was based on whether included studies accounted for patients with factors that would influence the predictability of RRT discontinuation, such as pre-existing chronic kidney disease (CKD) or prior RRT use in included cohorts. Disagreements were resolved through a discussion with a third author (OGR). The overall quality of evidence and certainty of outcome measures reported was further assessed according to the GRADE framework. A summary of findings’ table was prepared using the GRADEpro Guideline Development Tool (https://gradepro.org) for the pooled analysis. Full results of the risk of bias assessment are available in Additional file 3.

### Data analysis

Two-by-two tables of true/false positives/negatives were constructed from exposures and outcomes of weaning parameters reported from studies where available. When these quantities were not explicitly described, they were computed from other available data (e.g., sensitivity/specificity) where possible. When four or more studies reported on the same parameter in a sufficiently homogenous manner, the sensitivity and specificity were simultaneously pooled in a statistical meta-analysis using the bi-variate random effects method [36]. This model assumes that the correlated logit transformed values of the sensitivity and specificity are correlated and follow a bi-variate normal distribution, from which we can estimate not only simultaneous parameter estimates, but also a hierarchical summary receiver operating characteristic (AUROC) curve for the presented data. Review Manager (Version 5.3.5, Copenhagen, Denmark) was used to create the forest plots, while Stata (Version 14.2, College Station, Texas) was used to compute the bi-variate estimates.

## Results

### Search results

**Fig. 1:**
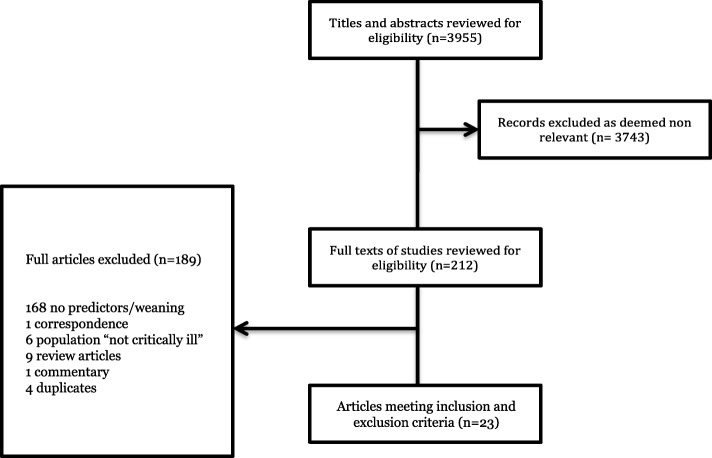
PRISMA flow diagram of retrieved and included records. Of the 23 included trials, 5 were abstracts and 18 were full text

Our initial search yielded 3031 citations and our updated search yielded an additional 924 citations. Twenty-three articles fulfilled all inclusion criteria (Fig. 1). This consisted of 18 full-text articles and five conference abstracts, representing two case-control studies, 15 retrospective cohort studies, and six prospective cohort studies (Table 1). There were no randomized controlled trials. All studies were published in English.

**Table 1:** Studies retrieved and parameters identified in a systematic review

Study	Study type	Population	#Patients with discontinuation of RRT	Quality (NOS)	Parameters identified	Definition of weaning success
Chen et al. [19]	Prospective cohort	Adult patients receiving CRRT	78	9	Urine output, plasma NGAL, serum Cr at discontinuation	Survival with no requirement for RRT within 7 days
Yoshida et al. [17]	Retrospective cohort	General ICU patients requiring RRT	38	8	Serum Cr, urine output, eGFR, kinetic eGFR at discontinuation	Survival off CRRT × 48 h, off IHD for 7 days
Jeon et al. [37]	Retrospective cohort	Adult patients with AKI on CRRT	517	9	Urine output, multivariate model at discontinuation	Survival with no re-initiation of RRT within 3 days
Itenov et al. [38]	Prospective cohort	Adult patients with AKI	719	8	Multivariate model	Survival with no re-initiation of RRT for 5 days
Kim et al. [28]	Prospective cohort	ICU patients weaned from CRRT	89	8	Serum cystatin C, plasma NGAL, urine output	Survival with no re-initiation of RRT for 14 days
Raurich et al. [27]	Retrospective cohort	ICU patients requiring CRRT who underwent weaning tests	67	9	Urine output with/without diuretics, multivariate model	Urine output recovered, RRT not required
Romero-Gonzalez et al. [39]	Retrospective cohort	Patients with AKI treated with CRRT	37	–	Urine output	Independence from RRT 14 days after discontinuation
Yang et al. [29]	Retrospective cohort	ICU patients weaned from CRRT, PIRRT, IHD	302	7	Serum cystatin C	Survival with no requirement for RRT 30 days after discharge
Yang et al. [30]	Prospective observational study	ICU patients who weaned from CRRT	102	7	Serum cystatin C	Survival at 60 days with Cr no more than 1.5X baseline
Aniort et al. [25]	Retrospective cohort	ICU patients receiving IHD for at least 7 days	67	8	Daily urine urea, eUrea, urine output	No requirement for further dialysis sessions during ICU stay
Katayama et al. [22]	Retrospective cohort	General ICU patients receiving CRRT	116	8	Urine output, Cr	Survival with no re-initiation of CRRT for 7 days
Han et al. [18]	Retrospective cohort	General ICU patients requiring CRRT	160	7	Multivariate model, Cr	Complete or partial recovery of AKI within 2 weeks
Kim et al. [40]	Prospective cohort	General ICU patients requiring RRT	89	4	Cystatin C-based eGFR	Survival with no re-initiation of RRT for 14 days
Viallet et al. [26]	Retrospective cohort	Adult patients who received CRRT, IHD or SLED and survived ICU stay	26	7	Urine output, urine Cr	Cessation of RRT for at least 15 days
Gleeson et al. [23]	Retrospective cohort	General ICU patients requiring RRT	67	6	Residual creatinine clearance	Not specified
Ohnuma et al. [41]	Retrospective cohort	General ICU patients requiring CRRT or IHD	109	5	Urine output	Free from RRT for 7 days after discontinuation
Frohlich et al. [20]	Retrospective cohort	General ICU patients requiring CRRT	53	6	2 h CrCl, Cr, urine output at discontinuation	Free from RRT for 7 days after discontinuation
Heise [42]	Retrospective cohort	Surgical ICU patients requiring CRRT	222	9	Multivariate model	Discharged from ICU with no further RRT during hospital stay
Zhang et al. [31]	Retrospective cohort	General ICU patients requiring CRRT	145	8	Serum cystatin C	Survivors who were not dependent on RRT
Solymos [43]	Retrospective cohort	General ICU patients requiring CRRT	23	6	2 h CrCl	Free from RRT for 5 days after discontinuation
Franzen et al. [44]	Retrospective cohort	Medical ICU patients requiring IHD	20	7	IHD ultrafiltration	No need for long-term RRT
Uchino et al. [21]	Prospective cohort	General ICU patients requiring CRRT	313	7	Urine output, Cr	Free from RRT for 7 days after discontinuation
Wu et al. [45]	Case control	Surgical ICU patients requiring CRRT or IHD	64	7	Multivariate model	Free from RRT for 30 days after discontinuation

### Study quality

**Fig. 2:**
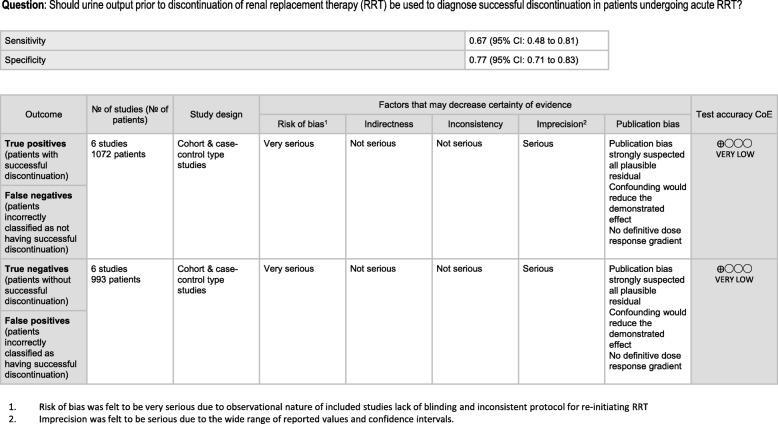
Summary of findings’ table for urine output prior to discontinuation of RRT

Study quality was generally rated as high for included observational studies, with a mean NOS score of seven (range 4–9) and no studies being rated as poor quality (see Additional file 3). Twenty studies were rated as high quality and two studies were rated as moderate quality. One conference abstract could not be rated due to insufficient information. Regarding the use of urine output as a predictor of successful RRT discontinuation, the overall certainty of the evidence was rated as very low given methodological limitations inherent to the included retrospective observational studies resulting in the risk of bias, and imprecision in the reported values (Fig. 2). There was consensus among authors as to the quality of the included studies.

### Markers of RRT weaning

A total of 16 variables predictive of RRT weaning and five multivariate models were assessed in 46 instances (Table 1). Weaning variables were grouped into four categories: conventional biochemical criteria (Table 2; *n* = 14; 29.8%), kidney biomarkers (Table 3; *n* = 13; 27.7%), physiologic criteria (Table 4, 5, 6; *n* = 13; 27.7%), and multivariate models (Table 7; *n* = 5; 14.9%). Urine output was the most commonly described variable. A total of nine studies described urine output criteria prior to RRT discontinuation and four studies described urine output after RRT discontinuation.

**Table 2:** Conventional biochemical criteria used to predict successful discontinuation of RRT

Test/parameter	Value/cut-off	Timing	RRT modality	Sn	Sp	OR	AUROC	Publication
Serum creatinine								
	299.68 umol/L	Initiation	CRRT	0.79	0.79		0.75	Yoshida et al. [17]
	Not specified	Initiation	CRRT				0.59	Han et al. [18]
	224 umol/L	Discontinuation	CRRT	0.72	0.77		0.76	Chen et al. [19]
	Per umol/L increase	Discontinuation	CRRT				0.48	Frohlich et al. [20]
	Not specified	Discontinuation	CRRT				0.64	Uchino et al. [21]
	Not specified	Discontinuation	CRRT				0.73	Katayama et al. [22]
2-h creatinine clearance								
	23 mL/min	12 h pre-stop	Not specified			1.11	0.82	Frohlich et al. [20]
Residual creatinine clearance								
	Not specified	24–48 h pre-stop	Not specified				0.90	Gleeson et al. [23]
Kinetic eGFR								
	20.58 mL/min/1.73 m^2^	Discontinuation	CRRT	0.71	0.92		0.87	Yoshida et al. [17]
eGFR								
	26.21 mL/min/1.73 m^2^	D1 post-stop	CRRT	0.71	0.85		0.83	Yoshida et al. [17]
24 h urine creatinine								
	> 5.2 mol/24 h	D0 post-stop	CRRT, SLED,	0.57	0.96		0.76	Viallet et al. [26]
		D1	IHD	0.75	0.88		0.86	
		D2		0.86	0.81		0.86	
Urine urea								
	> 148 mmol/L	Discontinuation	IHD, CRRT	0.65	0.90		0.82	Aniort et al. [25]
Daily urinary urea excretion								
	> 1.35 mmol/kg/day	Discontinuation	IHD, CRRT	0.89	0.97		0.96	Aniort et al. [25]

**Table 3:** Kidney biomarkers used to predict successful discontinuation of RRT

Test/parameter	Value/cut-off	Timing	RRT modality	Sn	Sp	OR	AUROC	Publication
Serum cystatin C								
	2.47 mg/L	Initiation	CRRT	0.95	0.54		0.75	Yang et al. [30]
	2.98 mg/L	ICU admission	CRRT	0.81	0.84	4.76	0.87	Zhang et al. [31]
	2.97 mg/L	Discontinuation	CRRT	0.80	0.58		0.71	Yang et al. [30]
	1.85 mg/L	Discontinuation	CRRT	0.76	0.63	0.29	0.74	Kim et al. [28]
	Not specified	Discontinuation	CRRT				0.74	Yang et al. [29]
Cystatin C-based eGFR								
	32.9 mL/min/1.73m^2^	Discontinuation	CRRT	0.65	0.76	1.25	0.75	Kim et al. [40]
NT-proBNP								
	> 15,767	Initiation	CRRT			0.54	0.58	Han et al. [18]
NGAL								
	Not specified	Discontinuation	CRRT	0.91	0.45		0.65	Kim et al. [28]
	403 ng/mL	Discontinuation	CRRT	0.91	0.61		0.81	Chen et al. [19]
	Not specified	Discontinuation	CRRT				0.66	Yang et al. [30]
IL-18								
	Not specified	Discontinuation	CRRT				0.60	Yang et al. [30]
IL-6								
	Not specified	Discontinuation	CRRT				0.55	Yang et al. [30]
Serum osteopontin								
	Not specified	Discontinuation	CRRT				0.61	Yang et al. [30]

**Table 4:** Urine output after discontinuation of RRT to predict successful weaning

Study	Cut-off value	# Patients	Sensitivity	Specificity	AUROC
Aniort et al. [25]	> 8.6 mL/kg/24 h	67	0.89	0.73	0.86
Gleeson et al. [23]	Not specified	157	Not estimable	Not estimable	0.87
Katayama et al. [22]	100 mL/day increase	213	Not estimable	Not estimable	0.81
Kim et al. [28]	> 1.26 mL/kg/h	110	0.60	0.67	0.67
Uchino et al. [21]	> 400 mL/day (no diuretics)	1006	0.46	0.81	0.85
Yoshida et al. [17]	> 1720 mL/24 h	52	0.68	0.86	0.78
Chen et al. [19]	> 715 mL/24 h	110	0.83	0.87	0.85
Jeon et al. [37]	> 191 mL/24 h	557	0.81	0.72	0.82
Romero-Gonzalez et al. [39]	> 720 mL/24 h	77	Not estimable	Not estimable	0.80
POOLED			0.66 (0.54, 0.77)	0.77 (0.71, 0.83) 0.74 (0.68, 0.79)	
			LR(−) 0.43	LR(+) 2.91

**Table 5:** Urine output prior to discontinuation of RRT to predict successful weaning

Study	Cut-off value	# Patients	Sensitivity	Specificity	AUROC
Raurich et al. [27]	> 178 mL in the 6 h post-discontinuation	86	0.90	0.89	0.91
Viallet [26]	> 2575 mL/24 h post-discontinuation	54	0.38	0.93	0.65
Yoshida et al. [17]	> 1709 mL/24 h post-discontinuation	52	0.76	0.79	0.77
Han et al. [18]	Not specified	160	Not estimable	Not estimable	0.63

**Table 6:** Effect of diuretic use on urine output test characteristics to predict successful discontinuation of RRT

Study	Cutoff Value	# Patients	Sensitivity (95% CI)	Specificity (95% CI)	AUROC (95% CI)
Jeon et al., diuretics [37]	191 mL/day	557	81.2 (77.6, 84.5)	71.6 (68.0, 75.0)	0.821 (0.797, 0.845)
Jeon et al., diuretics (oliguric) [37]	125 mL/day	619	72.1 (64.6, 78.8)	68.8 (61.3, 75.7)	0.745 (0.692, 0.798)
Raurich et al., no diuretics [27]	178 mL/6 h	42	–	–	0.73 (0.58, 0.89) before, 0.85(0.72, 0.99) after cessation
Raurich et al., diuretics [27]	178 mL/6 h	59	–	–	0.86 (0.76, 0.88) before, 0.94 (0.88, 1.0) after cessation
Uchino et al., no diuretics [21]	436 mL/day	335	46.5	80.9	0.845 (0.799, 0.883)
Uchino et al., diuretics [21]	2330 mL/day	194	–	–	0.671 (0.585, 0.750)
Yoshida et al., no diuretics [17]	1810 mL/day	22	61.5	77.8	0.71 (0.46, 0.88)
Yoshida et al., diuretics [17]	1720 mL/day	30	72.0	100.0	0.84 (0.64, 0.94)

**Table 7:** Multivariate models used to predict successful discontinuation of RRT

Test/parameter	Value/cut-off	Timing	#Patients	Modality	Sn	Sp	OR	AUROC	Publication
Multivariate									
	NT-proBNP, APACHE2, UO, Cr	At initiation	160	CRRT				0.70	Han et al. [18]
	Age, gender, UO, Cr	First 24 h of admission	719	CRRT				0.73	Itenov et al. [38]
	RRT duration, SOFA, oliguria, age	Discontinuation	64	CVVH/IHD				0.88	Wu et al. [45]
	Urine output, SOFA, #CRRT cycles	8 h post-stop	222	CRRT	0.74	0.74		0.80	Heise et al. [42]
	Urine output D0, kinetic eGFR D1	Discontinuation	38	CRRT	0.84	1.00		0.93	Yoshida et al. [17]

### Studies retrieved and parameters identified in a systematic review

There was a significant heterogeneity across studies in the definitions of “successful” RRT discontinuation and in the thresholds used to define weaning criteria. Most studies defined successful discontinuation as a specified period during which the patient did not receive further RRT; however, the periods specified varied and ranged from 3 days [37] to 60 days [30], with 7 days being the most frequently used (seven studies). Where multiple studies reported on the same variable, the threshold values with optimal predictive accuracy varied, as did the timing of measurement in relation to RRT discontinuation. For cystatin C, two studies measured values prior to RRT discontinuation [29, 31], and three measured values following discontinuation [28–30] (Table 3). The optimal threshold values ranged from 1.85 to 2.98 mg/L, respectively. The predictive accuracy of serum creatinine was assessed in two studies at RRT initiation [17, 18], and in four studies at the time of discontinuation [19, 20], with threshold levels ranging from 224 to 300 μmol/L (Table 2). Due to variation in the timing of measurement and threshold values, the pooled analysis was not feasible.

### Urine output prior to discontinuation of RRT to predict successful weaning

Urine output was reported in numerous studies, both before and after discontinuation. We felt that the timing of measurement relative to discontinuation (before or after) was the most clinically important characteristic affecting the heterogeneity of reporting; accordingly, we pooled data separately based on the timing of measurement. Urine output prior to RRT discontinuation was sufficiently homogenous across studies to perform pooled analysis (Fig. 2, Table 4). We found a pooled sensitivity of 66.2% (95% CI, 53.6 to 76.9), specificity of 73.6% (95% CI, 67.5 to 79.0), LR + 2.91, and LR − 0.43 for urine output (Fig. 2). Estimation of an optimal threshold to discriminate “successful” RRT discrimination was not feasible due to variation across studies, with thresholds ranging from 191 mL/24 h [37] to 1720 mL/24 h [17]. Overall certainty of evidence regarding this parameter was graded as very low (Fig. 2).

### Urine output after discontinuation of RRT to predict successful weaning

Urine output following RRT discontinuation was assessed in four studies and had a moderate AUC to predict successful RRT discontinuation (Table 5). Due to the low number of studies and heterogeneity, the pooled analysis was not feasible.

### Effect of diuretic use on urine output test characteristics to predict successful discontinuation of RRT

**Fig. 3:**
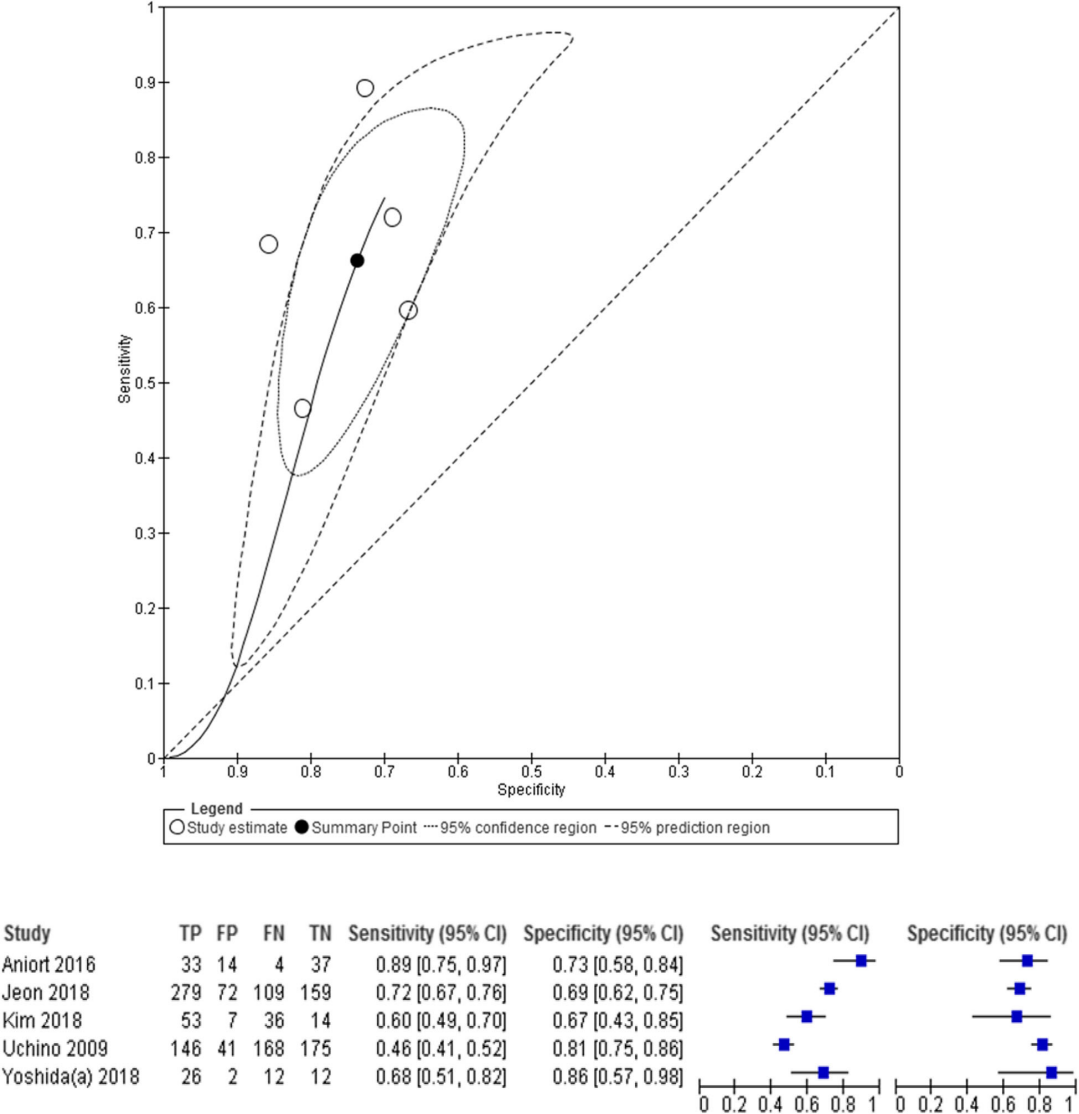
Pooled analysis for studies using urine output prior to discontinuation of RRT to predict successful weaning

Diuretic use was associated with successful RRT discontinuation in three studies [21, 37, 42], although test characteristics were not reported. The effect of a diuretic challenge on the predictive accuracy of urine output was assessed in four studies [17, 21, 27, 37] (Table 6). In two small studies [17, 27], there was a suggestion of improved predictive accuracy in patients who had received diuretics. However, a larger study [21] found that urine output was a less reliable predictor of successful discontinuation when diuretics were given and another study did not compare the accuracy of urine output with or without diuretics [37]. Diuretics used include furosemide as an intermittent dose [21, 27, 37, 42] or infusion [27, 37], spironolactone [37], thiazides [37], or other diuretics [21, 42]. No comparative analysis has been performed on the accuracy of urine output to predict RRT discontinuation among diuretic responders versus non-responders. Because the effect that concomitant diuretic exposure may have on the predictive capacity of urine output for RRT discontinuation remains uncertain, our pooled analysis does not adjust for diuretic-induced urine output (Fig. 3).

### Multivariate models used to predict successful discontinuation of RRT

Several multivariate models were studied and those which described the operative characteristics of variables, such as sensitivity, specificity, and AUROC are included in Table 7. The timing of these models varied, with one model integrating NT-proBNP, APACHE II score, urine output, and serum creatinine at RRT initiation (estimated AUROC of 0.70 [18]), and another model integrating age, sex, urine output, and serum creatinine during the first 24 h of admission (estimated AUROC of 0.73 [38]). The remaining three models included variables either at RRT discontinuation [17, 45] or at approximately 8 h after RRT discontinuation [42]. These models had good to excellent discrimination. In the study by Yoshida et al. [17], the model included urine output at day 0 (the day of CRRT discontinuation) and kinetic eGFR at day 1 (the day after CRRT discontinuation) and the AUROC was 0.93 for prediction of successful RRT discontinuation. None of the studies that included multivariable models have been externally validated.

### Secondary outcomes

Analysis of the relationships between reported weaning parameters and several secondary outcomes (new CKD, RRT duration, RRT use post-ICU discharge) was planned, but not carried out, as the available data made such analysis unfeasible (Additional file 4). New CKD and RRT use post-discharge were infrequently reported in included studies, and when reported were variably defined [27, 38, 39]. While RRT duration was specified in most studies, it was only analyzed in relation to weaning parameters in four instances [21, 22, 42, 45]. In three of these studies, RRT duration was included as a component of multivariate models used to predict successful discontinuation, as opposed to being the predicted endpoint. Given the infrequent reporting, heterogeneous definitions, and confounding effect of being both predictor and endpoint, we felt that meaningful inferences regarding these parameters could not be derived from the included studies and chose to omit the planned secondary analysis.

## Discussion

### Key findings

We found 16 unique parameters that have been evaluated for their ability to predict successful discontinuation of RRT and classified our findings into four categories: physiologic findings (i.e., urine output), conventional biochemical markers of kidney function (creatinine, urea, and estimates of GFR), kidney biomarkers (cystatin C and NGAL), and multivariate models integrating a variety of clinical and biochemical data.

Of all the variables described, urine output was the most commonly studied, with pooled sensitivities and specificities suggesting a modest (66.2% [95% CI, 53.6 to 76.9] and 73.6% [95% CI, 67.5 to 79.0]) predictive ability for successful RRT discontinuation for urine output. We found that there was substantial heterogeneity across studies in optimal thresholds for urine output, ranging from 191 mL/day [37] to over 1700 mL/day [17].

The effect of a diuretic challenge was variable among included studies, with some studies describing a decrease in the predictive ability of urine volume after diuretic administration [17, 27] and others describing superior discrimination following diuretic administration [21, 37]. The association of diuretic use with successful discontinuation of RRT [21, 37, 42] suggests that augmented diuresis may be an important management strategy to mitigate the need for re-initiation of RRT due to fluid accumulation following initial RRT discontinuation. A randomized controlled trial has previously demonstrated enhanced urinary volume and sodium excretion with infusion of furosemide in patients with resolving ARF; however, no overall improvement in renal outcome was seen, possibly due to infusion of fluids equal to the volume of diuresis in this trial which would have mitigated the potentially beneficial effects of a negative fluid balance [46].

At present, there is currently insufficient data to recommend a specific approach or identify a specific urine output threshold that may reliably predict successful RRT discontinuation. This is due to the fact that urine output was evaluated at different time points (i.e., preceding and following the exact timing of RRT discontinuation), with or without the actions of diuretics, and with cut-offs that varied greatly between studies. The pooled sensitivity and specificity represent the predictive ability of urine output in general, but the inability to determine an optimal threshold value based on available data currently limits the clinical utility of this parameter.

Kidney biomarkers have shown promise for prognostication in the setting of AKI and have been assessed in several studies evaluating RRT discontinuation and kidney recovery. Cystatin C was the most commonly studied, and though it appears to have a promising discrimination, due to substantial clinical heterogeneity in the timing of measurement and threshold values used in retrieved studies [28–30], data could not be pooled and definitive inferences on the accuracy of cystatin C measurement to predict successful RRT discontinuation could not be provided.

Numerous multivariate models have been proposed and generally shown good predictive ability (Table 7). Common features include measures of illness acuity (i.e., SOFA score or APACHE II score), urine output, and variables related to RRT duration (i.e., total days on RRT; number of RRT cycles). The timing of measurement of various parameters comprising a model is important. Among those models whose measurements are taken at the time of ICU admission or RRT initiation [18, 38], there is inherent confounding by a competing risk of death as a reason for RRT discontinuation, and there may be lower reliability to predict RRT in the intermediate term. Ideally, such models that integrate a spectrum of clinical information would have the capacity to inform and guide clinical decision-making on when to discontinue RRT. Those models that integrate variables taken at the time of or near the time of RRT discontinuation, along with other important time-varying variables (e.g., changes in acuity and non-kidney organ dysfunction) and which provide a standardized timeframe to assess re-initiation (with standardized criteria for re-initiation) are likely ideally suited to inform practice. Among the most promising models was the one described in the study by Yoshida et al., which combined the urine output on the day of discontinuation of RRT with the kinetic eGFR on the first-day post-discontinuation (AUROC 0.93) [17]. Importantly, these models would ideally undergo external validation to further assess performance and generalizability. Finally, each of these models utilized only 2 to 4 variables, never exceeding 10 events per variable in the multivariate models, thus minimizing the risk of model overfitting.

### Context with prior literature

Observational studies have shown that early re-initiation of RRT after a failed weaning attempt is associated with greater mortality, although it is unclear if this was more a surrogate of increased or evolving illness severity rather than non-recovery of kidney function and RRT independence [21]. A failed attempt to discontinue RRT may contribute to worsened or exacerbated physiologic profiles, uncertainty in drug dosing, and potentially re-exposing patients to the harmful sequelae of AKI, such as fluid accumulation, metabolic acidosis, and retention of metabolic waste.

Unlike weaning from mechanical ventilation, for which there are rigorously evaluated and published protocols [47, 48], the paucity of controlled trials to guide discontinuation of RRT has resulted in wide variations in practice [1, 49, 50], which may contribute to suboptimal care [51]. This may contribute to delays or premature discontinuation of RRT, which can have both patient-specific and health system-specific outcomes and resource implications.

Previous narrative reviews have suggested a weaning attempt of RRT in stable patients when the spontaneous urine output was > 400 mL/24 h and measured creatinine clearance was 15–20 mL/min [52], or when the urine output was > 30 mL/h and the 24-h urinary creatinine excretion was > 5.2 mmol/L [53]. Our study reaffirms the importance of urine output as a clinical marker to help guide RRT discontinuation; however, there is little evidence to determine an optimal threshold urine output value that can be reliably used by clinicians.

### Implications for future research

Our study highlights important avenues of future research and reveals significant knowledge gaps in the existing literature. Several markers that have been evaluated appear to have reasonable discrimination, such as daily urinary urea excretion (AUROC of 0.96) [25], and kinetic eGFR (a method developed to reflect true GFR in situations where the serum creatinine is changing dynamically; AUROC of 0.87 in two different studies [17, 24]). However, these have generally be evaluated in small, isolated, retrospective studies and there is a need for replication in larger prospective cohorts before reliable inferences can be made. In addition, further work is needed to understand optimal urine output thresholds, the ideal measurement intervals to predict a successful RRT discontinuation, and further validation across clinical care settings and case-mix.

The predictive ability of urine output could be further augmented to aid clinical decision-making by a combination of patient-specific variables (i.e., age, CKD status) with time-varying and dynamic variables (i.e., daily SOFA score, kinetic GFR, RRT duration) [17, 45]. This would be an important advance in updating clinical practice guidelines with recommendations that are more detailed and clinically prescriptive for clinicians.

### Strengths and limitations

Our review identifies and synthesizes a wide array of physiologic and biochemical markers of weaning success for acute renal replacement therapy. The strengths of our study include the peer-reviewed comprehensive search strategy and a rigorous methodology as outlined in the PRISMA guideline.

Despite these strengths, there are some important limitations in the studies retrieved that warrant consideration. First, although the overall quality of included studies was felt to be high, all data were derived from observational studies, as no randomized trials assessing liberation from RRT were found. Second, many different markers have been studied; however, most have only been evaluated in a single study, have not been replicated, and have not been externally validated. Where markers have been assessed in multiple studies, we found significant heterogeneity in the thresholds used to define positive or negative results, the timing of measurement with respect to the discontinuation of RRT, and in the operational definition used for weaning success. This limited the ability to generalize results and provide a pooled estimate of predictive ability for markers that have been studied in multiple cohorts.

Finally, we were unable to carry out a planned analysis of secondary endpoints such as new CKD, RRT duration, and RRT use post-ICU discharge. With the exception of RRT duration, these endpoints were infrequently reported and none of the included studies related these endpoints to parameters used to predict RRT discontinuation in a way that could be meaningfully aggregated. Subgroup analyses were also planned [34], stratified by age, RRT modality, and CKD status; however, we found that these subgroups were not well described and were heterogeneously defined across studies and thus did not permit further subgroup analysis.

## Conclusions

Our systematic review identified 16 variables for the prediction of successful RRT discontinuation. Where multiple studies reported on the same parameter, the timing of measurement and threshold values used were heterogeneous, making pooled analysis not feasible for most. Urine output prior to discontinuation of RRT was the most-studied variable to predict RRT discontinuation (pooled sensitivity and specificity of 66.2% and 73.6%); however, an optimal threshold value was not determined due also to heterogeneity across retrieved studies. Future work should focus on refinement of a urinary output threshold value and the development and validation of a clinical prediction tool, incorporating urine output with other static and dynamic clinical variables, to better guide clinicians on when to discontinue RRT in ICU settings.

## Supplementary information

**Additional file 1.** PRISMA Checklist. PRISMA Checklist for DOnE_RRT.

**Additional file 2.** Search Strategy. Complete search strategy for DOnE_RRT.

**Additional file 3.** Risk of Bias Assessment. Complete numerical breakdown of.

**Additional file 4.** Secondary Outcomes Table. Table recording planned secondary outcomes as reported in retrieved studies.

## Data Availability

All data generated or analyzed during this study are included in this published article.
