# Supplementary material for: Determining the optimal time for liberation from renal replacement therapy in critically ill patients: a systematic review and meta-analysis (DOnE RRT)
Source: Crit Care. 2020 Feb 13;24:50. doi: 10.1186/s13054-020-2751-8 (PMC7020497; doi:10.1186/s13054-020-2751-8)
Supplement: Supplementary file 2 — Additional file 2. Search Strategy. Complete search strategy for DOnE_RRT. [file 13054_2020_2751_MOESM2_ESM.docx]

**Additional File 2: Search strategy**

Database: Ovid MEDLINE(R) Epub Ahead of Print, In-Process & Other Non-Indexed Citations, Ovid MEDLINE(R) Daily and Ovid MEDLINE(R) 1946 to Present

Date Searched: 30 August 2017

Date of Update Search: 05 April 2019

Strategy:

1 Acute Kidney Injury/th (9383)

2 Hemodiafiltration/ (2109)

3 Kidney Diseases/th (6976)

4 Renal Dialysis/ (85284)

5 Renal Replacement Therapy/ (4626)

6 CRRT*.tw,kf. (1245)

7 (dialys* or h?emodialys*).tw,kf. (146517)

8 (h?emo diafiltrat* or h?emodiafiltrat* or h?emo filtrat* or h?emofiltrat*).tw,kf. (6503)

9 (renal replacement adj2 (support* or treat* or therap*)).tw,kf. (10648)

10 RRT*.tw,kf. (3796)

11 or/1-10 [Combined MeSH & text words for renal replacement therapy] (180593)

12 Coronary Care Units/ (4279)

13 Critical Care/ (47004)

14 Critical Illness/ (23314)

15 Intensive Care Units/ (45948)

16 Multiple Organ Failure/ (10142)

17 Respiratory Care Units/ (582)

18 (cardiac adj2 (department* or unit* or ward*)).tw,kf. (2177)

19 critical care*.tw,kf,jw. (104608)

20 critical* ill*.tw,kf. (41994)

21 ((department* or unit* or ward*) adj2 intensive*).tw,kf. (96100)

22 ((department* or unit* or ward*) adj2 respiratory).tw,kf. (1634)

23 ICU*.tw,kf. (46948)

24 intensive care*.tw,kf,jw. (137230)

25 intensivist*.tw,kf. (2896)

26 (multi* organ* adj1 (d#s function* or d#sfunction* or fail*)).tw,kf. (12811)

27 (multi* system* adj1 (d#s function* or d#sfunction* or fail*)).tw,kf. (304)

28 or/12-27 [Combined MeSH & text words for critical care] (291588)

29 Critical Care Outcomes/ (16)

30 *Predictive Value of Tests/ (1524)

31 Recovery of Function/ (43193)

32 *Time Factors/ (1854)

33 *Treatment Outcome/ (6386)

34 Withholding Treatment/ (10773)

35 (ceas* or cessation*).tw,kf. (87522)

36 (dis-continu* or discontinu*).tw,kf. (113193)

37 (function* adj2 (recover* or restor*)).tw,kf. (42113)

38 liberat*.tw,kf. (23220)

39 (predict* adj5 recover*).tw,kf. (5667)

40 (recover* adj2 renal).tw,kf. (3186)

41 stop*.tw,kf. (118147)

42 terminat*.tw,kf. (109121)

43 transition*.tw,kf. (339649)

44 wean*.tw,kf. (43218)

45 (withdraw* or withhold*).tw,kf. (121066)

46 or/29-45 [Combined MeSH & text words for discontinuation] (1000991)

47 and/11,28,46 [Combined concepts of RRT, critical care & discontinuation] (1094)

48 limit 47 to yr="1990-Current" (1055)

49 exp animals/ not humans/ (4530947)

50 48 not 49 [Excluded animal studies] (1046)

51 remove duplicates from 50 (1007)

Database: Ovid Embase 1988 to 2017 Week 35

Date Searched: 1 September 2017

Date of Update Search: 05 April 2019

Strategy:

1 acute kidney failure/th (8416)

2 exp continuous renal replacement therapy/ (6578)

3 hemodiafiltration/ (2381)

4 *hemodialysis/ (33017)

5 *kidney disease/th (2744)

6 *renal replacement therapy/ (14348)

7 CRRT*.tw,kw. (2241)

8 (dialys* or h?emodialys*).tw,kw. (159217)

9 (h?emo diafiltrat* or h?emodiafiltrat* or h?emo filtrat* or h?emofiltrat*).tw,kw. (8160)

10 (renal replacement adj2 (support* or treat* or therap*)).tw,kw. (16220)

11 RRT*.tw,kw. (6091)

12 or/1-11 [Combined Emtree & text words for renal replacement therapy] (184207)

13 exp coronary care unit/ (7039)

14 critical illness/ (24659)

15 intensive care/ (103887)

16 intensive care unit/ (117048)

17 multiple organ failure/ (30486)

18 (cardiac adj2 (department* or unit* or ward*)).tw,kw. (3198)

19 critical care*.tw,kw,jx. (167127)

20 critical* ill*.tw,kw. (57787)

21 ((department* or unit* or ward*) adj2 intensive*).tw,kw. (125947)

22 ((department* or unit* or ward*) adj2 respiratory).tw,kw. (2565)

23 ICU*.tw,kw. (86930)

24 intensive care*.tw,kw,jx. (183505)

25 intensivist*.tw,kw. (4978)

26 (multi* organ* adj1 (d#s function* or d#sfunction* or fail*)).tw,kw. (19115)

27 or/13-26 [Combined Embase & text words for critical care] (447992)

28 convalescence/ (40902)

29 critical care outcome/ (91)

30 *predictive value/ (4663)

31 *time factor/ (293)

32 *treatment outcome/ (16823)

33 treatment withdrawal/ (14677)

34 (ceas* or cessation*).tw,kw. (95034)

35 (dis-continu* or discontinu*).tw,kw. (147645)

36 (function* adj2 (recover* or restor*)).tw,kw. (53153)

37 liberat*.tw,kw. (18001)

38 (predict* adj5 recover*).tw,kw. (7532)

39 (recover* adj2 renal).tw,kw. (4057)

40 stop*.tw,kw. (147008)

41 terminat*.tw,kw. (104798)

42 transition*.tw,kw. (315834)

43 wean*.tw,kw. (46485)

44 (withdraw* or withhold*).tw,kw. (138686)

45 or/28-44 [Combined Emtree & text words for discontinuation] (1067992)

46 and/12,27,45 [Combined concepts of RRT, critical care & discontinuation] (2476)

47 limit 46 to yr="1990-Current" (2458)

48 exp animal/ not human/ (3274115)

49 47 not 48 [Excluded animal studies] (2445)

50 remove duplicates from 49 (2386)

Database: Wiley Cochrane Library

Date Searched: 1 September 2017

Date of Update Search: 08 April 2019

Strategy:

#1 [mh ^"Acute Kidney Injury"/TH] 291

#2 [mh ^Hemodiafiltration] 228

#3 [mh ^"Kidney Diseases"/TH] 214

#4 [mh ^"Renal Dialysis"] 4147

#5 [mh ^"Renal Replacement Therapy"] 199

#6 CRRT*:ti,ab,kw 120

#7 (dialys* or haemodialys* or hemodialys*):ti,ab,kw 11920

#8 ("haemo diafiltrat*" or "haemo filtrat*" or haemodiafiltrat* or haemofiltrat* or "hemo diafiltrat*" or "hemo filtrat*" or hemodiafiltrat* or hemofiltrat*):ti,ab,kw 1086

#9 ("renal replacement" near/2 (support* or treat* or therap*)):ti,ab,kw 1228

#10 RRT*:ti,ab,kw 258

#11 {or #1-#10} 13155

#12 [mh ^"Coronary Care Units"] 155

#13 [mh ^"Critical Care"] 1830

#14 [mh ^"Critical Illness"] 1563

#15 [mh ^"Intensive Care Units"] 2206

#16 [mh ^"Multiple Organ Failure"] 364

#17 [mh ^"Respiratory Care Units"] 14

#18 (cardiac near/2 (department* or unit* or ward*)):ti,ab,kw 213

#19 "critical care*":ti,ab,kw,so 12744

#20 "critical* ill*":ti,ab,kw 4846

#21 ((department* or unit* or ward*) near/2 intensive*):ti,ab,kw 11555

#22 ((department* or unit* or ward*) near/2 respiratory):ti,ab,kw 178

#23 ICU*:ti,ab,kw 5926

#24 "intensive care*":ti,ab,kw,so 16027

#25 intensivist*:ti,ab,kw 161

#26 ("multi* organ*" near/1 ("dis function*" or disfunction* or "dys function*" or dysfunction* or fail*)):ti,ab,kw 1181

#27 ("multi* system*" near/1 ("dis function*" or disfunction* or "dys function*" or dysfunction* or fail*)):ti,ab,kw 4

#28 {or #1-#27} 40482

#29 [mh "Critical Care Outcomes"] 0

#30 [mh ^"Predictive Value of Tests" [mj]] 31

#31 [mh ^"Recovery of Function"] 4437

#32 [mh ^"Time Factors" [mj]] 18

#33 [mh ^"Withholding Treatment"] 300

#34 [mh ^"Treatment Outcome" [mj]] 323

#35 (ceas* or cessation*):ti,kw 7012

#36 ("dis-continu*" or discontinu*):ti,kw 1197

#37 (function* near/2 (recover* or restor*)):ti,ab,kw 6821

#38 liberat*:ti,ab,kw 469

#39 (predict* near/5 recover*):ti,ab,kw 467

#40 (recover* near/2 renal):ti,ab,kw 184

#41 stop*:ti,kw 1357

#42 terminat*:ti,ab,kw 7085

#43 transition*:ti,ab,kw 5165

#44 wean*:ti,ab,kw 2286

#45 (withdraw* or withhold*):ti,kw 15417

#46 {or #29-#45} 45254

#47 #11 and #28 and #46 546

#48 #11 and #28 and #46 Publication Year from 1990 to 2017 521

Other Source: Conference Proceedings Citation Index (1990-present) via Web of Science Core Collection

Date Searched: 3 October 2017

Date of Update Search: 08 April 2019

Strategy:

TS=("acute kidney injur*"' OR CRRT* OR haemodia* OR haemofiltrat* OR hemodial* OR hemofiltrat* OR "renal replacement therap*" OR RRT) AND TS=("critical care" OR "critical* ill*" OR ((cardiac OR intensive) AND (department* OR unit* OR ward*)) OR ICU OR "intensive care") AND TS=(ceas* OR cessation* OR discontinu* OR liberat* OR outcome* OR predict* OR recover* OR stop* OR terminat* OR transition* OR wean* OR withdraw* OR withhold*)

Limit to 2015 – 2017 (41)

Other Source: ClinicalTrials.gov

Date Searched: 10 October 2017

Date of Update Search: 08 April 2019

Strategy:

Advanced Search >

Other Terms: (ceas* OR cessation OR discontin* OR liberat* OR predict* OR restor* OR recover* OR restor* OR stop* OR terminat* OR wean* OR withdraw* OR withhold*)

Intervention/Treatment: (CRRT OR dialysis OR hemodialysis OR hemodiafiltrat* OR "renal replacement" OR RRT) (4)
