# Supplementary material for: Determining the optimal time for liberation from renal replacement therapy in critically ill patients: a systematic review and meta-analysis (DOnE RRT)
Source: Crit Care. 2020 Feb 13;24:50. doi: 10.1186/s13054-020-2751-8 (PMC7020497; doi:10.1186/s13054-020-2751-8)
Supplement: Supplementary file 4 — Additional file 4. Secondary Outcomes Table. Table recording planned secondary outcomes as reported in retrieved studies. [file 13054_2020_2751_MOESM4_ESM.docx]

Additional File 4

| Study (First author, year) | Population Included | RRT Modality | Secondary endpoint(s) reported | | | Secondary endpoints assessed in relation to predictive factors |
| --- | --- | --- | --- | --- | --- | --- |
|  |  |  | Duration of RRT | New CKD | RRT Utilization post-ICU discharge |  |
| **Chen 2019** | All adult patients with AKI receiving RRT | CRRT | Yes (hours) | No | No | N/A |
| **Romero-Gonzalez 2018** | Mixed ICU population, Italy | CRRT | No | No | Yes | N/A |
| **Jeon 2018** | Adult patients with AKI | CRRT | Yes (days) | No | No | N/A |
| **Itenov 2018** | Adult patients with AKI, no ESRD | Unspecified | No | Reported % recovered prior renal function | No | N/A |
| **Yoshida 2018** | All adult patients who received RRT | CRRT | Yes (days) | No | No | N/A |
| **Kim 2018** | ICU patients weaned successfully | CRRT | Yes (days) | No | No | N/A |
| **Raurich 2018** | ICU patients requiring RRT who underwent weaning tests | CRRT | Yes (days) | No | Yes | N/A |
| **Yang 2017 (1)** | ICU patients weaned from RRT | CRRT, PIRRT, IHD | # sessions | No | Excluded | N/A |
| **Yang 2017 (2)** | ICU patients who weaned from RRT | CRRT, IHD | # sessions, days of RRT | No | No | N/A |
| **Aniort 2016** | Medical ICU patients | IHD, CRRT | # sessions, days of RRT | No | No | N/A |
| **Katayama 2016** | General ICU patients | CRRT | Yes (days) | No | No | RRT duration |
| **Han 2016** | General ICU patients | CRRT | “duration of weaning” | No | No | N/A |
| **Kim 2016** | General ICU patients | Unspecified | No | No | No | N/A |
| **Viallet 2016** | Adult survivors, medical ICU | CRRT, IHD SLED | Yes (days) | No | No | N/A |
| **Gleeson 2015** | General ICU patients | Unspecified | No | No | No | N/A |
| **Ohnuma 2013** | General ICU patients | CRRT, IHD | No | No | No | N/A |
| **Frohlich 2012** | General ICU patients | CRRT | No | No | No | N/A |
| **Heise 2012** | Surgical ICU patients | CRRT | Yes (#cycles) | Yes | No | #cycles part of multivariate model |
| **Zhang 2012** | General ICU patients | CRRT | No | No | No | N/A |
| **Solymos 2011** | Adult patients | CRRT | No | No | No | N/A |
| **Franzen 2010** | Mixed ICU population | CRRT | Yes (days) | Yes | No | N/A |
| **Uchino 2009** | Adult patients with AKI- multicenter | CRRT | First CRRT period (days) | No | No | Length of first CRRT period included in multivariate regression |
| **Wu 2008** | Surgical ICU patients with AKI | Unspecified | Yes (days) | No | No | Length of dialysis included in multivariate regression |

Secondary outcomes reported in included trials
